# *Fitm2* is required for ER homeostasis and normal function of murine liver

**DOI:** 10.1016/j.jbc.2023.103022

**Published:** 2023-02-15

**Authors:** Laura M. Bond, Ayon Ibrahim, Zon W. Lai, Rosemary L. Walzem, Roderick T. Bronson, Olga R. Ilkayeva, Tobias C. Walther, Robert V. Farese

**Affiliations:** 1 Department of Molecular Metabolism, Harvard T.H. Chan School of Public Health, Boston, Massachusetts, USA; 2 Department of Cell Biology, Harvard Medical School, Boston, Massachusetts, USA; 3 Harvard T.H. Chan Advanced Multi-omics Platform, Department of Molecular Metabolism, Harvard T. H. Chan School of Public Health, Boston, Massachusetts, USA; 4 Department of Poultry Science and Graduate Faculty of Nutrition, Kleberg Animal & Food Science Center, Texas A&M University, College Station, Texas, USA; 5 Rodent Histopathology Core, Harvard Medical School, Boston, Massachusetts, USA; 6 Division of Endocrinology, Metabolism, and Nutrition, Department of Medicine, Duke Molecular Physiology Institute, Sarah W. Stedman Nutrition and Metabolism Center, Duke University School of Medicine, Durham, North Carolina, USA; 7 Cell Biology Program, Sloan Kettering Institute, Memorial Sloan Kettering Cancer Center, New York, New York, USA; 8 Broad Institute of Harvard and MIT, Cambridge, Massachusetts, USA; 9 Howard Hughes Medical Institute, Boston, Massachusetts, USA

**Keywords:** FITM2, liver, acyl-CoA, endoplasmic reticulum, lipid metabolism, ANOVA, analysis of variance, ER, endoplasmic reticulum, FIT2, fat storage–inducing transmembrane protein 2, FIT-LKO, FIT2 knockout mice, HDL, high-density lipoprotein, HFD, high-fat diet, IFNγ, interferon γ, PC, phosphatidylcholine, PE, phosphatidylethanolamine, TG, triglyceride

## Abstract

The endoplasmic reticulum (ER)–resident protein fat storage–inducing transmembrane protein 2 (FIT2) catalyzes acyl-CoA cleavage *in vitro* and is required for ER homeostasis and normal lipid storage in cells. The gene encoding FIT2 is essential for the viability of mice and worms. Whether FIT2 acts as an acyl-CoA diphosphatase *in vivo* and how this activity affects the liver, where the protein was discovered, are unknown. Here, we report that hepatocyte-specific *Fitm2* knockout (FIT2-LKO) mice fed a chow diet exhibited elevated acyl-CoA levels, ER stress, and signs of liver injury. These mice also had more triglycerides in their livers than control littermates due, in part, to impaired secretion of triglyceride-rich lipoproteins and reduced capacity for fatty acid oxidation. We found that challenging FIT2-LKO mice with a high-fat diet worsened hepatic ER stress and liver injury but unexpectedly reversed the steatosis phenotype, similar to what is observed in FIT2-deficient cells loaded with fatty acids. Our findings support the model that FIT2 acts as an acyl-CoA diphosphatase *in vivo* and is crucial for normal hepatocyte function and ER homeostasis in the murine liver.

The endoplasmic reticulum (ER) is the major cellular site of lipid synthesis and production of cell surface and secreted proteins. The protein fat storage–inducing transmembrane protein 2 (FIT2) has emerged as an important determinant of ER homeostasis in cells. FIT1 and FIT2 genes were identified as targets of the transcription factor PPARα in the murine liver (1). Both FIT1 and FIT2 are ER-resident proteins with six putative transmembrane segments and ∼50% amino acid sequence similarity (2). Murine FIT1 and FIT2 have different tissue expression patterns: FIT1 is expressed mainly in skeletal and cardiac muscle, and FIT2 is ubiquitously expressed, with the highest levels in adipose tissue (1).

The molecular functions of FIT proteins have been somewhat enigmatic. FIT2 was initially implicated in lipid metabolism and, in particular, lipid droplet (LD) biogenesis (1, 3). Overexpression of FIT2 in murine liver results in increased lipid storage in hepatocytes, and this finding was replicated in a variety of cell types (1). In cells, FIT2 was localized to the ER and found at sites of LD biogenesis (4). FIT2 binds neutral lipids and was hypothesized to partition neutral lipids into a storage pool (5). The FIT2 gene is essential in worms and mice (6, 7), highlighting the importance of FIT2 function. Tissue-specific deletions of *Fitm2* in mice revealed crucial functions in adipocyte differentiation, enterocyte function, and pancreatic β-cells (7, 8, 9). In humans, homozygous *FITM2* deficiency causes deafness–dystonia syndrome (10, 11), and human FIT2 is required for cancer cell fitness during exposure to interferon γ (IFNγ) (12). The diverse deletion phenotypes show that FIT2 is crucial for life and highlight that FIT2 deficiency manifests differently in different biological systems.

FIT2 is required for normal cellular ER homeostasis. It was identified as a putative lipid phosphate phosphatase enzyme by homology searches (13), and it has acyl-CoA diphosphatase activity *in vitro*, utilizing a variety of acyl-CoA substrates to generate acyl 4′-phosphopantetheine and adenosine-3′,5′-bisphosphate products (14). This enzymatic activity is critical to preserving cellular ER homeostasis, as FIT2 deficiency in mammary carcinoma cells and yeast results in ER dilation and whorls, ER stress, and reduced LD biogenesis capacity (6, 14). Consistent with these data, FIT2 orthologs in yeast, *SCS3* and *YFT2*, were implicated in ER homeostasis (15, 16).

In the current study, we sought to determine whether FIT2 acts as an acyl-CoA–cleaving enzyme and functions in ER homeostasis *in vivo* by deleting *Fitm2* in murine hepatocytes. We studied the effects of FIT2 deficiency on hepatic ER and lipid homeostasis by analyzing the phenotypes of mice fed chow or high-fat diets (HFD). Our results demonstrate the necessity of FIT2 for ER homeostasis *in vivo* and provide insights into its physiological functions in this tissue.

## Results

### Generation of hepatocyte-specific FIT2 knockout mice

**Figure 1:**
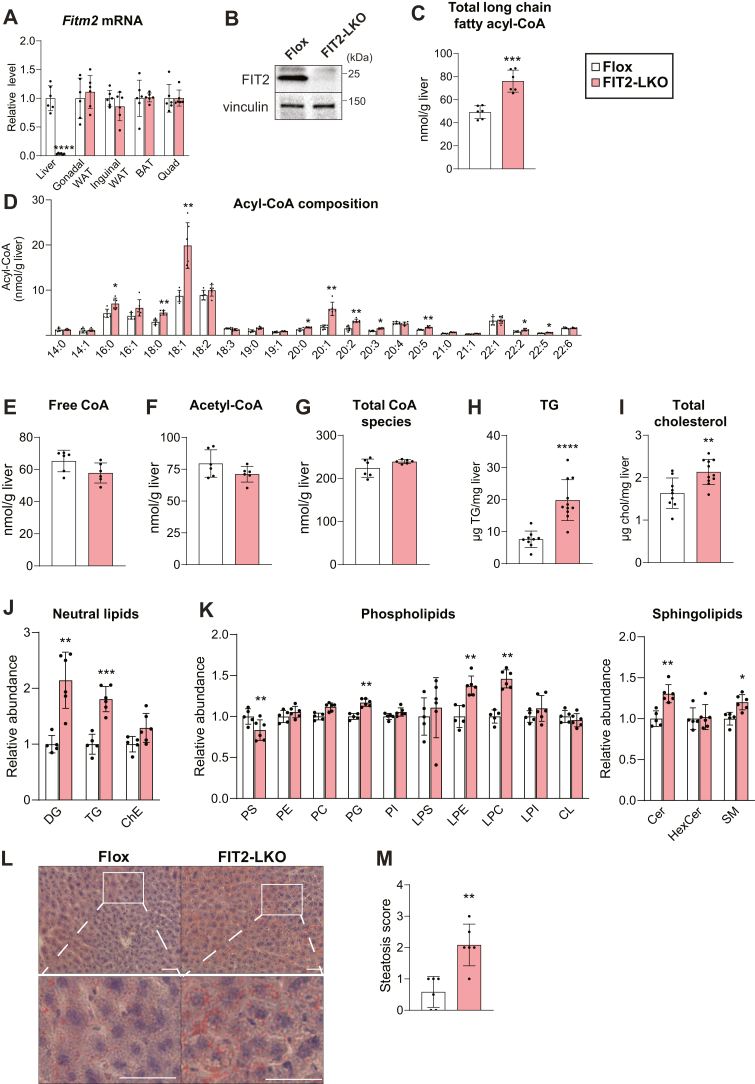
**Hepatocyte-specific FIT2-LKO mice have altered hepatic lipid composition.***A*, hepatocyte-specific FIT2 knockout results in the near loss of *Fitm2* transcript in liver (n = 9–11/genotype) but does not alter *Fitm2* transcript levels in adipose tissue and muscle (n = 6/genotype) as measured by qRT-PCR. *B*, immunoblotting indicates loss of FIT2 protein in liver tissue. *C*–*G*, MS-based measurements of CoA and CoA derivatives in livers of Flox and FIT2-LKO mice (n = 6/genotype). *C*, FIT2-LKO livers contain elevated levels of total long-chain fatty acids (≥14 carbon length). *D*, Amounts of individual long-chain acyl-CoA species in livers of Flox and FIT2-LKO mice. Levels of hepatic free CoA (*E*), acetyl-CoA (*F*), and total CoA-containing species (*G*) are not significantly altered between Flox and FIT2-LKO mice. Hepatic triglyceride levels (*H*) and cholesterol levels (*I*) are elevated in FIT2-deficient livers (n = 9–11/genotype). Lipidomic analyses of levels of neutral lipids (*J*) and phospholipids (*K*) in male FIT2-LKO mice (n = 6/genotype). Representative images (*L*) and steatosis scoring (*M*) of *Oil Red* O staining indicates more neutral lipid content in FIT2-LKO livers than in Flox livers. (n = 6/genotype). Scale bar = 50 μm. Data represents mean ± SD. Statistical significance for was evaluated with unpaired Student two-tailed *t* test for (*A*–*K*) and a Mann–Whitney *U* test for nonparametric data in (*M*). ∗*p* < 0.05, ∗∗*p* < 0.01, ∗∗∗*p* < 0.001, ∗∗∗∗*p* < 0.0001. Cer, ceramide; CerG1, glucosylceramide; ChE, cholesterol ester; CL, cardiolipin, DAG, diacylglycerol; FIT2, fat storage–inducing transmembrane protein 2; FIT-LKO, FIT2 knockout mice; L, lyso; PC, phosphatidylcholine; PE, phosphatidylethanolamine; PG, phosphatidylglycerol, PI, phosphatidylinositol; PS, phosphatidylserine; SM, sphingomyelin; TG, triglyceride.

To investigate the function of FIT2 in murine liver, we generated hepatocyte-specific FIT2 knockout mice (FIT2-LKO) using Cre-loxP technology. We crossed *Fitm2*^*loxP/loxP*^ (floxed) mice with mice expressing Cre recombinase under the control of the albumin promoter, leading to the deletion of exon 1 of *Fitm2* (Fig. S1*A*). Hepatic *Fitm2* transcript and protein levels, as measured by immunoblot analysis, were reduced by 95% (Fig. 1, *A* and *B*). Mass spectrometry of liver lysates also demonstrated the loss of FIT2 protein in FIT2-LKO livers (Table S1). We confirmed that Cre recombinase expression was restricted to liver and not found in muscle or adipose tissue of FIT2-LKO mice (data not shown). Consistently, *Fitm2* mRNA levels were normal in skeletal muscle, brown adipose tissue, gonadal, and inguinal white adipose tissue (Fig. 1*A*). To determine whether hepatocyte FIT2 deletion resulted in compensation by FIT1, we measured FIT1 protein levels by mass spectrometry. FIT1 was present in skeletal muscle but was not detected in livers of either floxed control or FIT2-LKO mice, consistent with reports that FIT1 protein is not expressed in murine liver (Table S1) (1).

### FIT2 deficiency alters acyl-CoAs and other hepatic lipids

To determine whether FIT2 functions as an acyl-CoA diphosphatase in hepatocytes, we tested whether FIT2 deficiency leads to the accumulation of acyl-CoA substrates. We measured acyl-CoA and CoA levels in control and FIT2-LKO livers (Table S2) and found that long-chain fatty acyl-CoA levels were elevated by ∼60% (Fig. 1*C*). Specifically, we found marked increases in the levels of several species of acyl-CoAs, including the monounsaturated fatty acyl-CoAs 18:1 and 20:1 and lesser increases in several minor species (Fig. 1*D*). In contrast, levels of free CoA, acetyl-CoA, and total CoA-containing species were normal (Fig. 1, *E*–*G* and Table S2). These data are consistent with previous findings that FIT2 hydrolyzes long-chain unsaturated fatty acyl-CoAs but not short-chain acyl-CoA species or free CoA (14).

Because FIT2 functions in fatty acid metabolism, we also investigated changes in hepatic lipids. In contrast to reduced triglyceride (TG) storage in cells with FIT2 deficiency (1, 3, 14), hepatic triglyceride content was unexpectedly elevated ∼2.5-fold, and cholesterol content was increased ∼20% in both male and female mice (Figs. 1, *H* and *I* and S1*B*). Mass spectrometry–based lipidomic analyses of the livers of control and FIT2-LKO mice corroborated the elevated triglyceride levels and revealed a twofold increase in diacylglycerol levels (Fig. 1*J*). Levels of the major phospholipids, phosphatidylcholine (PC) and phosphatidylethanolamine (PE), were not altered, but levels of several other phospholipids (*e.g.*, lysophosphatidylcholine, lysophosphatidylethanolamine, and phosphatidylglycerol) were slightly elevated, and phosphatidylserine levels were modestly reduced (Fig. 1*K*). Levels of ceramide and sphingomyelin were modestly elevated (Fig. 1*K*).

We analyzed lipid deposition in histological sections with Oil Red O and H&E staining (Figs. 1*L* and S1*C*). Neutral lipid deposition was low in both genotypes and agreed with minimal lipid accumulation noted under chow feeding. However, steatosis scores were greater in FIT2-LKO mice than in floxed control mice (Fig. 1*M*). The increase in hepatic triglycerides was accompanied by elevated liver weights in both males and females (Fig. S1*D*). Body weights were not altered between the genotypes after chow feeding (Fig. S1*E*). Collectively, these results show that liver-specific FIT2 deficiency increases neutral lipid (*i.e.*, TG) storage under conditions of chow feeding.

### Loss of FIT2 disrupts ER homeostasis and causes liver injury

**Figure 2:**
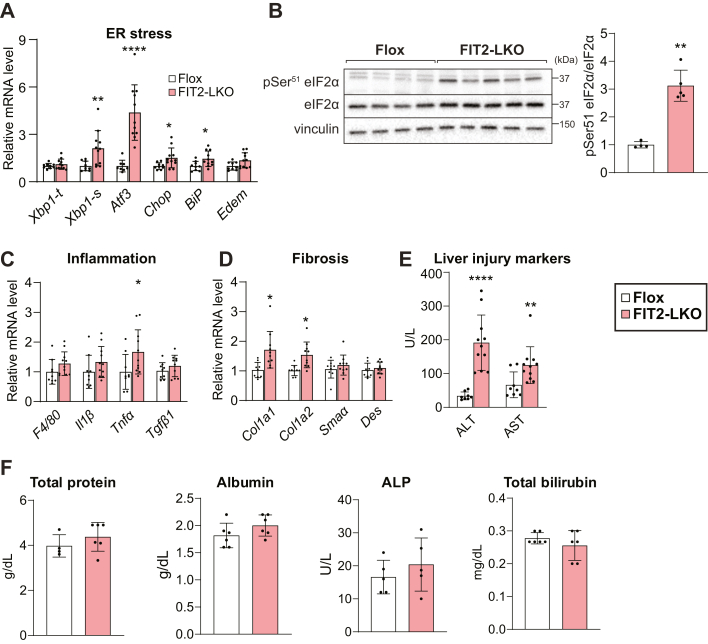
**FIT2-LKO mice exhibit increased ER stress and liver injury.***A*, gene expression analyses demonstrate that FIT2-LKO mice have elevated transcript levels of chaperones and transcription factors associated with ER stress compared to chow-fed Flox mice (n = 9–11). *B*, immunoblotting indicates more phosphorylation of eIF2α in FIT2-LKO livers than in Flox littermates (n = 3–4). *C*, FIT2-LKO livers do not show much evidence for inflammation, as indicated by transcript levels of macrophage markers and cytokines (n = 9–11/genotype). *D*, FIT2-LKO livers do not show evidence for substantial fibrosis (n = 9–11/genotype). *E*, FIT2-LKO mice exhibit liver injury. Levels of circulating alanine transaminase (ALT) and aspartate transaminase (AST) are higher in FIT2-LKO mice than in Flox controls (n = 9–11). *F*, plasma albumin, total protein, alkaline phosphatase (ALP) and bilirubin are unaltered in FIT2-LKO mice (n = 6). Data represent mean ± SD. Statistical significance for (*A*, *C* and *D*) was evaluated with unpaired Student two-tailed *t* test for parametric data and a Mann–Whitney *U* test for nonparametric data. Statistical significance for (*B*) was evaluated with unpaired Student’s two-tailed *t* test. For (*E* and *F*), two-way analysis of variance with Šidák correction was used. ∗*p* < 0.05, ∗∗*p* < 0.01, ∗∗∗*p* < 0.001, ∗∗∗∗*p* < 0.0001. ER, endoplasmic reticulum; FIT2, fat storage–inducing transmembrane protein 2; FIT-LKO, FIT2 knockout mice.

Since FIT2 is crucial for maintaining ER homeostasis in cultured mammalian and yeast cells, we examined ER morphology and function in FIT2-LKO mice. FIT2-LKO mice exhibited modest ER stress, reflected in increased mRNA levels of transcription factors (*e.g.*, *Xbp1*, A*tf3*, *Chop*) and chaperones (*e.g.*, *BiP*) associated with the unfolded protein response in FIT2-LKO livers (Fig. 2*A*). Phosphorylation of eIF2α was ∼threefold greater in the livers of FIT2-LKO mice than in floxed control littermates (Fig. 2*B*). Analysis of hepatocyte ER structure by EM showed that hepatic FIT2 deficiency did not cause detectable ER dilation (Fig. S2, *A* and *B*). Total ER content was also apparently unaltered as quantified by EM (Fig. S2*C*) and proteomic analysis of ER markers (Fig. S2*D*). Consistent with these measurements, the levels of PC and PE, major phospholipids constituents of the ER, were not altered (Fig. 1*K*).

Because inflammation often accompanies ER stress, we assessed hepatic immune cell infiltration and cytokine production. Consistent with chow feeding eliciting minimal inflammation and negligible fibrosis, transcript levels of these markers were low in both genotypes (data not shown). However, transcript levels of macrophage markers and cytokines trended higher in FIT2-deficient livers (Fig. 2*C*). Also, some markers of fibrosis and transcript levels of pro- and anti-apoptotic genes were elevated (Figs. 2*D* and S2, *E* and *F*).

Despite minimal evidence for inflammation or apoptosis, FIT2-deficient livers displayed evidence of injury. Plasma levels of transaminases alanine transaminase and aspartate transaminase were elevated seven- and twofold, respectively (Fig. 2*C*). Plasma markers of synthetic liver function (total protein, albumin) and cholestasis (bilirubin, alkaline phosphatase) were normal (Fig. 2*D*).

### Impaired TG secretion and reduced fatty acid oxidation capacity contribute to TG accumulation in chow-fed FIT2-LKO mice

**Figure 3:**
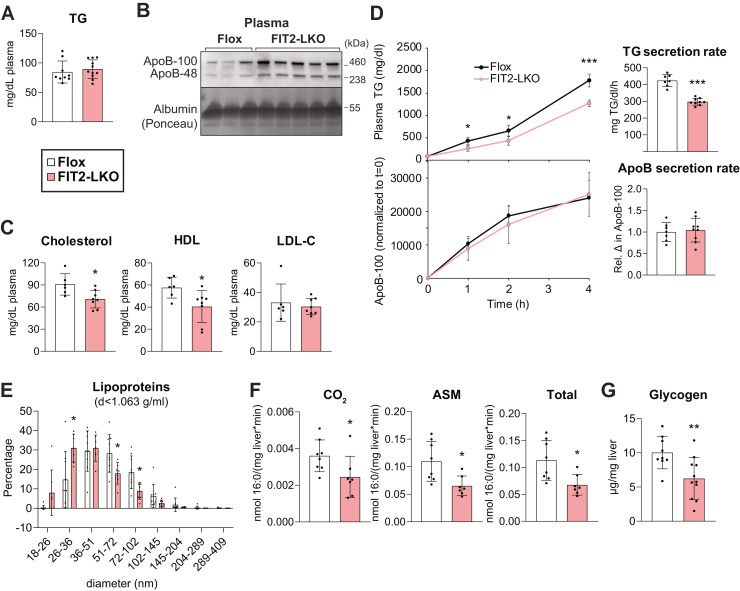
**Chow-fed FIT2-LKO mice exhibit alterations in TG metabolism that contribute to steatosis.***A*, steady-state plasma TG levels are unaltered in Flox and FIT2-LKO mice (n = 9–11/genotype). *B*, steady-state levels of plasma apoB-100 and apoB-48 are higher in plasma of FIT2-LKO mice than in Flox controls. Mice were fasted 2 h before sacrifice in (*A*) and (*B*). *C*, plasma cholesterol levels were decreased in the FIT2-LKO; specifically, HDL-C was reduced, but LDL-C remained the same. *D*, FIT2-LKO mice exhibit reduced rates of hepatic TG secretion but unaltered rates of hepatic apoB secretion. Plasma was collected before (t = 0) and 1, 2, and 4 h after intravenous administration of polaxomer-407, a lipoprotein lipase inhibitor. Plasma TG was assayed biochemically. Relative plasma apoB-100 protein levels were determined by quantification of apoB-100 band intensity on immunoblots (Fig. S3*A*). *E*, dynamic light scattering measurements indicate that lipoprotein (density < 1.063) particle diameter (number distribution) is reduced in FIT2-LKO mice compared to Flox mice (n = 7–9/genotype; mice were fasted 4-h before sacrifice). *F*, FAO capacity is reduced in FIT2-LKO liver lysates (n = 5–8/genotype). *G*, hepatic glycogen levels are reduced in male FIT2-LKO mice compared to Flox mice (n = 9–11/genotype). Data represent mean ± SD. Statistical significance for (*A*, *C*, *F*, and *G*) was evaluated with unpaired Student two-tailed *t* test. For (*D*, *left*), repeated-measures analysis of variance (ANOVA) was used and for (*D*, *right*), unpaired Student two-tailed *t* test was used. For (*E*), two-way ANOVA with Šidák correction was used. ∗*p* < 0.05, ∗∗*p* < 0.01, ∗∗∗*p* < 0.001. ER, endoplasmic reticulum; FIT2, fat storage–inducing transmembrane protein 2; FIT-LKO, FIT2 knockout mice; TG, triglyceride.

To elucidate the causes of hepatic TG accumulation found in chow-fed FIT2-LKO mice, we analyzed pathways that influence TG levels. We evaluated very low-density lipoprotein (VLDL) secretion since this pathway depends on ER phospholipid and protein composition and occurs in the ER lumen (17, 18), the proposed location of the catalytic residues of FIT2 (2, 14). Steady state plasma levels of TG were unaltered, and levels of the primary protein component of VLDL, apolipoprotein (apo) B, were increased in FIT2-LKO animals (Fig. 3, *A* and *B*). LDL-cholesterol levels were also similar in both genotypes (Fig. 3*C*). However, we found that TG secretion by the liver was reduced by ∼30% in the FIT2-LKO mice (Fig. 3*D*). In contrast, secretion of apoB, quantified by immunoblotting, was similar between genotypes (Figs. 3*D* and S3*A*). Proteomic analyses of the livers indicated that protein levels of apoB and the microsomal TG transfer protein, required for lipidation of apoB, were similar among genotypes (Fig. S3*B*). Since circulating apoB was consistently elevated and TG secretion was reduced in FIT2-LKO mice, we hypothesized that FIT2-LKO hepatocytes secrete smaller VLDL particles. To test this, we determined the size distribution of particles recovered from the *d* < 1.063 g/ml fraction of plasma. We found an increase in the total percentage of particles ≤51 nm in diameter (88% v. 72%) and a decrease in particles ≥72 nm in diameter (12% v. 27%) in FIT2-LKO plasma, consistent with this hypothesis. Among factors required for optimal lipoprotein secretion, lysophosphatidylcholine acyltransferase 3 (LPCAT3, also known as MBOAT5) catalyzes the formation of PC with arachidonyl-CoA on the ER lumen, which promotes lipoprotein secretion (17, 19). However, levels of 20:4-PC were not reduced in FIT2-LKO livers (Fig. S3*C*), indicating that changes in PC acyl-chain composition are unlikely to be the cause of the secretion phenotype of FIT2-LKO livers.

We also investigated whether impaired fatty acid oxidation contributes to the increased steatosis of chow-fed FIT2-LKO. Testing mitochondrial fatty acid oxidation was compelling as the deafness–dystonia syndrome reported in humans with *FITM2* mutations is reminiscent of a similar disorder, Mohr-Tranebjaerg syndrome, that is caused by defects in mitochondrial function (20, 21). Liver lysates from FIT2-LKO mice had a reduced capacity to produce acid-soluble metabolites and CO_2_ by oxidizing fatty acids (Fig. 3*F*). This was not due to a reduction in transcript or protein levels of fatty acid oxidation enzymes or mitochondrial content as assessed by oxidative phosphorylation gene expression, protein levels, and mitochondrial DNA content (Fig. S3, *D*–*I*). Impaired fatty acid oxidation appeared to alter fuel utilization and increased reliance on glucose oxidation in FIT2-LKO. Electron microscopy revealed a marked decrease in glycogen in hepatocytes of FIT2-LKO mice (Fig. S2*A*), which was corroborated by a biochemical assay for hepatic glycogen (Fig. 2*G*).

### HFD worsens liver injury in FIT2-LKO mice

To further test the role of FIT2 in liver lipid and ER homeostasis, we challenged mice with an HFD (42% kcal from fat). We hypothesized that this diet would result in sustained acyl-CoA overexposure and exacerbate the phenotypes found with standard chow feeding. With respect to general parameters, FIT2-LKO mice unexpectedly gained less weight than control littermates during the 11-week feeding study (Fig. S4*A*). The reduced weight gain was due to reduced body fat, with both gonadal and inguinal white adipose tissues showing a reduced mass in FIT2-LKO mice (Fig. S4*B*). Weekly food consumption was similar among genotypes suggesting that increased energy expenditure led to the lower body weight phenotype (Fig. S4*C*). We did not investigate this aspect of the phenotype further, but hepatic injury may have resulted in more energy expenditure.

**Figure 4:**
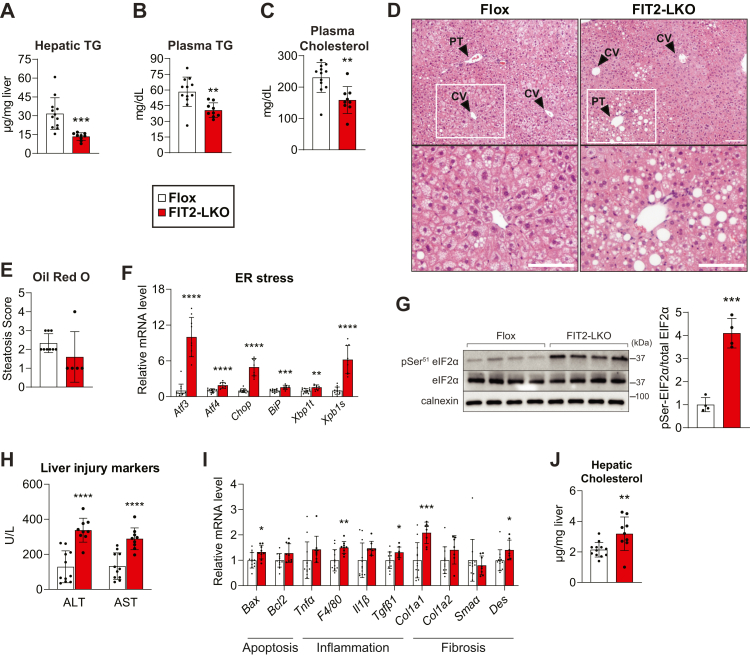
**High-fat diet (HFD) feeding exacerbates ER stress and liver injury in FIT2 liver-specific KO mice.** Levels of hepatic TG (*A*), plasma TG (*B*), and plasma cholesterol (*C*) are decreased in FIT2-LKO mice after 11 weeks of HFD feeding. *D*, representative images of H&E staining of livers from Flox and FIT2-LKO mice after HFD feeding. Scale bar = 50 μm. *E*, steatosis scoring of Oil Red O staining of Flox and FIT2-LKO mice given HFD challenge (n = 5–9/genotype). *F*, RT-qPCR studies show that under HFD, FIT2-LKO mice have increased expression of ER stress genes. *G*, this is supported by increased phosphorylation of the UPR protein eIF2α, as shown with western blotting (n = 4/genotype). *H*, after HFD challenge, FIT2-LKO mice exhibit exacerbated liver injury, as shown by measurement of plasma alanine transaminase and aspartate transaminase (ALT and AST). *I*, this phenotype presents with relatively minor changes in the expression of gene markers for apoptosis, inflammation, or fibrosis. *J*, FIT2-LKO mice had increased levels of hepatic cholesterol. Data represent mean ± SD. N = 9 to 12/genotype, unless otherwise noted above for specific experiments. Statistical significance was evaluated with unpaired Student two-tailed *t* test for (*A*–*C*, *G* and *J*). For (*E*, *F* and *I*), statistical significance was evaluated with unpaired Student’s two-tailed *t* test for parametric data and a Mann–Whitney *U* test for nonparametric data. For (*H*), Two-way analysis of variance with Šidák correction was used. ∗*p* < 0.05, ∗∗*p* < 0.01, ∗∗∗*p* < 0.001, ∗∗∗∗*p* < 0.0001. CV, central vein; ER, endoplasmic reticulum; FIT2, fat storage–inducing transmembrane protein 2; FIT-LKO, FIT2 knockout mice; PT, portal triad; TG, triglyceride.

In contrast to the results with a chow feeding, hepatic TG content was ∼50% less in the FIT2-LKO mice fed the HFD than in controls (Fig. 4*A*). This was accompanied by reductions in plasma TGs and cholesterol (Fig. 4, *B* and *C*). The decreased hepatic lipid content was visible in H&E-stained liver tissue sections and scoring of Oil Red O staining (Figs. 4, *D* and *E* and S4*D*). Examination of the lipid deposition revealed that Flox control mice exhibited extensive centrilobular microsteatosis; in contrast, the FIT2-LKO livers exhibited predominately macrosteatosis and fat accumulation localized to the periportal zone. Consistent with the findings of reduced lipid levels, FIT2-LKO livers also showed a decrease in the expression of genes of *de novo* lipogenesis (Fig. S4*E*). FIT2-LKO also exhibited reduced liver glycogen levels (Fig. S4*F*), consistent with the hypothesis that they utilize carbohydrates for fuel. HFD-fed FIT2-LKO mice exhibited elevated plasma ketone bodies (Fig. S4*G*), although they showed little to no differences in the expression of fatty acid oxidation or oxidative phosphorylation-related genes (Fig. S4*H*). These metabolic changes were accompanied by a ∼20% reduction in total long-chain acyl-CoA levels in FIT2-LKO compared with controls, driven largely by decreases in 16:1 acyl-CoA and 18:1 acyl-CoA (Fig. S4, *I*–*J* and Table S3). Free CoA levels were increased and acetyl-CoA levels were decreased in HFD-fed FIT2-LKO mice (Fig. S4, *K*–*L* and Table S3). An explanation for reduced long-chain acyl-CoA levels in HFD-fed FIT2-LKO mice (*versus* the analogous chow feeding studies) is not immediately apparent but may reflect the complexity of CoA metabolism under HFD conditions, as reflected in the change in the other CoA pools.

As with chow feeding, FIT2-LKO mice showed evidence of hepatic ER stress and injury. Levels of both mRNA transcripts and phosphorylated protein markers of the unfolded protein response were increased in the FIT2-LKO mice to an even greater degree than under standard chow feeding (Fig. 4, *F* and *G*). Moreover, plasma ALT and AST markers of liver injury were further increased in the FIT2-LKO mice after the HFD challenge (Fig. 4*H*). Although apoptotic, inflammation, and fibrosis often accompany such severe ER stress and liver damage, these markers were not substantially altered between genotypes (Fig. 4*I*).

## Discussion

Previous data showed that the ER-resident FIT2 has acyl-CoA diphosphatase activity *in vitro* and is important for maintaining ER homeostasis in human and yeast cells (14). We now show that hepatic deficiency of FIT2 results in increased acyl-CoA levels *in vivo*, which are linked to increased ER stress and signs of liver injury, as manifested by elevated circulating transaminase levels. The latter findings for hepatic FIT2 deficiency were exacerbated with HFD feeding. Although it is uncertain whether humans with FIT2 deficiency exhibit similar hepatocyte defects (10, 11), our studies highlight the crucial importance of FIT2 in lipid and ER homeostasis *in vivo*.

In contrast to what was found in FIT2-deficient cultured cells (14), we found no gross morphological changes in the ER or ER whorls in hepatocytes of FIT2-LKO mice. A possible explanation for the absence of ER morphology changes could be that increased autophagic flux cleared such structures, particularly since autophagy ameliorates liver damage in certain contexts (22). Consistent with this notion, FIT2 interacts genetically with autophagic pathways; FIT2 deletion sensitizes Renca cancer cells to cell death from IFNγ and inactivation of autophagy reverses this phenotype (12).

The reduction in TG storage under HFD feeding conditions is similar to what has been reported for FIT2 deficiency in cells that have been cultured with excess fatty acids (1, 14). However, unexpectedly, chow-fed FIT2-LKO mice accumulated neutral lipids and TGs in hepatocytes. The modest level of steatosis in chow-fed FIT2-LKO mice may be at least partially explained by reductions in TG secretion and fatty acid oxidation capacity. With respect to TG secretion, our results are consistent with the hypothesis that FIT2 deficiency impairs the loading of nascent lipoproteins with TGs. Since FIT2 is hypothesized to act on the luminal leaflet of the ER, FIT2 deficiency may lead to acyl-CoA accumulation at this leaflet and interfere with the lipidation of the nascent apoB particles in the ER lumen. Similarly, changes in ER phospholipids can have marked effects on TG secretion, although we did not find changes in 20:4-PC, which has been directly implicated in this process (17, 23, 24).

The reduction in fatty oxidation capacity in lysates of the FIT2-LKO livers was substantial and may also contribute to the TG accumulation in chow-fed mice. We found no differences in mitochondrial content, gene expression, or protein levels, suggesting that FIT2 deficiency adversely affects fatty acid oxidation through an as-yet-unknown mechanism. Of note, altered mitochondrial biology is consistent with the human FIT2 deficiency phenotype; patients with FIT2 mutations present with deafness–dystonia symptoms similar to those afflicted with Mohr-Tranebjaerg syndrome, which is caused by defects in mitochondrial function (20, 21).

Our findings highlight that the phenotypes of ER stress and lipid accumulation with FIT2 deficiency can be dissociated. We found ER stress to be a consistent observation in all our studies of FIT2 deficiency in cells and mice, on either chow or HFD. In contrast, the lipid accumulation phenotype in mice appears to be contextual and depends on the dietary status. This supports the hypothesis that lipid storage phenotypes are a secondary consequence and not a primary role for FIT2 in LD formation (14). In support of this, loss of FIT2 in pancreatic β cells is accompanied by ER stress, and FIT2 deficiency resulted in several fold increased levels of tissue ceramides (9). We also found ceramide levels were increased in FIT2-LKO livers but to a lesser extent (∼30%) (Fig. 1*K*).

The mechanism of how FIT2 deficiency results in ER stress is unclear. Most proximally, the defects associated with FIT2 deficiency are likely due either to accumulation of its substrates (*e.g.*, unsaturated acyl-CoAs) or to deficiency of its products (*i.e.*, 3′,5′-ADP and acyl 4′-phosphopantetheine). Although this remains uncertain at present, FIT2 activity is clearly important for the health of cells. Interestingly, human FIT2 protects Renca cancer cells from IFNγ effects (12). Additionally, high intratumoral levels of *FITM2* expression correlate with decreased survival in human patients with hepatocellular carcinoma (3). Thus, FIT2 inhibitors may be useful to sensitize specific cancer cells to targeted chemotherapies. Continued studies to elucidate the consequences of FIT2 deficiency will be essential for unraveling why FIT2 activity is so crucial to cell health and, hopefully, for finding therapies for humans suffering from the consequences of FIT2 deficiency.

## Experimental procedures

### Animals

*Fitm2*^*flox/flox*^ mice (028832, Jackson Laboratory) (8) were crossed with mice expressing Cre recombinase under control of the albumin promoter (B6.Cg-Tg(Alb-Cre)21MGN/J). Mice were housed in the Harvard School of Public Health animal care facility and maintained on a 12-h light–dark cycle. Mice had free access to water and food unless specified otherwise. Mice were weaned to a standard rodent chow diet (PicoLab Rodent Diet 20 #5053). For HFD studies, animals were weaned to a chow diet and then fed TD.88137 (42% calories from fat) for 11 weeks starting at 7 weeks of age. For food intake measurements, animals were individually caged 1 week prior to data collection. All mice were male and fasted 2 h prior to sacrifice unless specified otherwise. Mice were euthanized with isoflurane, blood was collected *via* cardiac puncture, and tissues were collected. All *in vivo* mouse experiments were conducted in accordance with the protocol approved by the Institutional Animal Care and Use Committee at the Harvard University.

### Quantitative PCR

For gene expression studies, tissues were homogenized in Qiazol using a Bead Mill Homogenizer (VWR), RNA was isolated using RNeasy kit (Qiagen), and cDNA was synthesized using an iSCRIPT cDNA synthesis kit (Bio-Rad). qPCR was performed using Power SYBR Green PCR Master Mix kit (Applied Biosystems). Primer sequences are listed in Table S4.

For the assessment of mitochondrial DNA (mtDNA) content, total DNA was prepared using the QIAmp DNA mini Kit (Qiagen). mtDNA was amplified using primers for *Co1* and *Nd1* and was normalized to genomic DNA using primers amplifying H19. Primer sequences are listed in Table S4.

### Immunoblotting

Livers were homogenized in RIPA buffer (Cell Signaling Technology), supplemented with complete mini EDTA-free protease inhibitor (Sigma Aldrich) and PhosSTOP phosphatase inhibitor (Sigma-Aldrich) using a Bead Mill Homogenizer (VWR). Protein concentrations were measured using a DC Protein Assay (Bio-Rad). Proteins were incubated at 60°C for 15 min in 4× Laemmli sample buffer (Bio-Rad). Liver lysate protein of 20 to 40 μg was separated by SDS-PAGE and transferred to a polyvinylidene fluoride membrane. The membrane was blocked with TBS-T containing 5% nonfat dry milk for 1 h at room temperature and then incubated overnight at 4 °C in primary antibody. Membranes were washed in TBS-T, incubated in secondary antibody, washed with TBS-T, and visualized using SuperSignal Chemiluminescent Substrate (Thermo Scientific). Band intensity was measured using ImageJ software. Antibodies used include total eIF2a (Cell Signaling Technology #9722, 1:1000, in 5% BSA), phosphor-Ser51-eIF2a (Cell Signaling Technology #9721, 1:1000), vinculin (Cell Signaling Technology #4650, 1:1000), ApoB (Abcam ab31992, 1:1000), and FIT2 (a generous gift from David Silver’s laboratory (1), 1:1000)

### Acyl-CoA measurements

Cellular and liver acyl-CoA esters were analyzed using a method based on a report by Magnes *et al.* (25) that relies on the extraction procedure described by Deutsch *et al.* (26). The CoAs were further purified by solid phase extraction as described by Minkler *et al.* (27). The acyl CoAs were analyzed by flow injection analysis using positive electrospray ionization on Xevo TQ-S, triple quadrupole mass spectrometer (Waters) employing methanol/water (80/20, v/v) containing 30 mM ammonium hydroxide as the mobile phase. Spectra were acquired in the multichannel acquisition mode monitoring the neutral loss of 507 amu (phosphoadenosine diphosphate) and scanning from m/z 750 to 1060. Heptadecanoyl CoA was employed as an internal standard. The endogenous CoAs were quantified using calibrators prepared by spiking cell or liver homogenates with authentic CoAs (Sigma) having saturated acyl chain lengths C_0_-C_18._. Corrections for the heavy isotope effects, mainly ^13^C, to the adjacent m + 2 spectral peaks in a particular chain-length cluster were made empirically by referring to the observed spectra for the analytical standards. Statistical significance was evaluated with multiple unpaired *t* tests followed by Benjamini, Krieger, and Yekutieli FDR correction of 1% for multiple hypothesis testing, and q values are reported.

### Proteomics

Liver (∼20 mg) was homogenized in 800 μl of PBS (supplemented with complete mini EDTA-free protease inhibitor (Sigma Aldrich) and 5 mM EDTA) using a Bead Mill Homogenizer (VWR). The extraction of proteins was performed as described (28). Mass spectrometry data were analyzed by MaxQuant software version 1.5.2.8 (29) using the following setting: oxidized methionine residues and protein N-terminal acetylation as variable modification, cysteine carbamidomethylation as fixed modification, first search peptide tolerance 20 ppm, and main search peptide tolerance 4.5 ppm. Protease specificity was set to trypsin with up to two missed cleavages allowed. Only peptides longer than six amino acids were analyzed, and the minimal ratio count to quantify a protein is 2. The false discovery rate (FDR) was set to 5% for peptide and protein identifications. Database searches were performed using the Andromeda search engine integrated into the MaxQuant environment (30) against the UniProt-mouse database containing 54,185 entries (December 2018). “Matching between runs” algorithm with a time window of 0.7 min was utilized to transfer identifications between samples processed using the same nanospray conditions. Protein tables were filtered to eliminate identifications from the reverse database and common contaminants. Fold changes of proteins were calculated by comparing the mean area of log2 intensities between replicates of different genotypes. Statistical significance was calculated using a Student *t* test followed by Benjamini–Hochberg FDR correction of 5% for multiple hypothesis testing.

### Lipidomics

Liver (∼100 mg) was homogenized in 1 ml of PBS using a Bead Mill Homogenizer (VWR). Lipids were extracted, according to the Folch method (31). Lysis volume was normalized to starting tissue material. The organic fraction containing extracted lipids was subjected to liquid chromatography/tandem mass spectrometry analysis as described in (28). Mass spectrometry data analysis was performed using LipidSearch version 4.1 SP (Thermo Fisher Scientific). The results were exported to R-Studio where quality control was performed using pairwise correlations between replicates, a principal component analysis comparing sample groups, as well as retention time plot analysis to verify elution clustering within lipid classes. All identified lipids were included for subsequent analyses if they fulfilled the following LipidSearch-based criteria: (1) reject equal to zero, (2) main grade A or main grade B and a *p* value of <0.01 for at least three replicates, and (3) no missing values across all samples. Statistical significance was calculated using a Student *t* test followed by a Holm-Sidak test to correct for multiple comparisons.

### Histology

Livers were collected and fixed in formalin overnight at 4 °C. Livers were sectioned and stained by the Rodent Histopathology Core at Harvard Medical School. Frozen sections were used for Oil Red O staining, which were unbiasedly scored for steatosis by a histopathologist (28). Paraffin-embedded tissue was used for H&E staining. Sections were imaged on a ZEISS light microscope.

### Electron microscopy

Mice were anesthetized with isoflurane and then perfused with 10 ml of PBS followed by 10 ml of 2.5% glutaraldehyde, 2.5% paraformaldehyde in 0.1 M sodium cacodylate buffer (pH 7.4). Liver pieces of 1 to 2 mm were fixed in the fixative overnight, washed several times in 0.1 M cacodylate buffer, osmicated in 1% osmium tetroxide/1.5% potassium ferrocyanide (final solution) for 3 h, and followed by several washes of dH_2_O. 1% uranyl acetate in maleate buffer was added for 1 h and then washed several times with maleate buffer (pH 5.2). This was followed by a graded cold ethanol series up to 100%, which is changed 3× over 1 h, followed by propylene oxide, changed 3× over 1 h. The sample was then placed in ½ and ½ propylene oxide with a plastic mixture including a catalyst overnight. The following day, samples were polymerized in Taab 812 Resin (Marivac Ltd) at 60°C for 24 to 48 h. Sections of 80 nm were cut with a Leica ultracut microtome, picked up on 100 mesh formvar/carbon-coated copper grids, stained with 0.2% lead citrate, and viewed, and imaged with a JEOL 1200X electron microscope equipped with an MP 2k CCD camera. For ER dilation quantification, three images (representative of ER dilation for that animal) were selected per mouse, and the distance across the ER lumen (bilayer center-to-bilayer center) was measured using ImageJ. At least 25 lumen measurements were calculated per image and averaged to provide the representative ER dilation for that mouse. For total ER quantification, three images (representative of ER content for that a) were selected per mouse. Using ImageJ, the total cell area was traced and calculated (nucleus excluded due to variability in nuclear size), and the ER was manually traced. ER content was calculated as nm ER length divided by μm^2^ available cell area. Four flox and seven FIT2-LKO animals were assessed using this method, and the data depict the average and standard deviation of these biological samples.

### TG and apoB secretion measurements

Mice were fasted for 4 h and injected intravenously with 1000 mg/kg body weight with the lipoprotein lipase inhibitor, Poloxamer-407. Tail vein blood was collected at t = 0, 1, 2, and 4 h. Plasma was supplemented with complete mini EDTA-free protease inhibitor (Sigma Aldrich) and snap frozen. Plasma TG was measured using Infinity TG kit (Thermo Scientific). ApoB-100 protein levels were measured by immunoblotting, as described earlier. At 1 h, the sample was diluted 1:5. At 2 and 4 h, they were diluted 1:10. 1.5 μl of plasma (or 1.5 μl of diluted plasma) was heated at 95°C for 5 min in 2× denaturing sample buffer. Plasma samples from each time point were run on the same gel. Equal loading was confirmed by visualization of albumin with Ponceau staining. To compare between gels, a sample from each time point was run on the t = 0 gel (Fig. S3*A*).

### VLDL particle size measurements

Mice were fasted 4 h prior to sacrifice; 2 × 10 μl was removed for density profiling by isopycnic ultracentrifugation (32). For each plasma sample, the d < 1.063 g/ml fraction was prepared by ultracentrifugation in a Beckman Coulter Optima MAX-XP benchtop ultracentrifuge in an MLA-55 rotor (18 h × 172,301*g* at 14 °C). This fraction contains virtually all of the apo-B100 in the plasma (33). Lipoprotein particle diameters were determined by dynamic light scattering analysis with a Microtrac Series 150 Ultrafine particle analyzer fitted with a flexible conduit-sheathed probe tip (UPA-150; Microtrac) (34, 35). Raw particle-size distributions from number distributions were converted to population percentiles, which were used to calculate the median particle diameter for each decile of lipoprotein size distribution.

### *Ex vivo* fatty acid oxidation assay

Mice were fasted for 4 h and euthanized with isoflurane. Liver was collected and processed *via* Dounce homogenization in 2 ml of sucrose-Tris-EDTA buffer as detailed (36). Liver homogenates were centrifuged at 450*g* for 10 min at 4 °C. Supernatants were collected and incubated for 1 h in the presence of fatty acid oxidation substrate (300 μM palmitic acid with 0.4 μCi 1-14C-palmitic acid). 1 mM rotenone, an inhibitor of oxidative phosphorylation, was used as a control. The radioactivity of trapped CO_2_ and acid-soluble metabolites were measured using a liquid scintillation counter. Fatty acid oxidation rates were calculated as [(counts per minute-blank)/reaction mixture specific activity]/g tissue.

### Liver biochemical assays

Liver (∼50 mg) was homogenized in 500 μl of lysis buffer (250 mM sucrose, 50 mM Tris HCl, pH 7.4). Lipids were extracted using a modified Bligh and Dyer method and solubilized in 0.1% Triton-X-100 by sonication (three rounds of 2 s at 30 mA). TG and cholesterol were quantified using Infinity Triglyceride and Cholesterol Reagents (Thermo Scientific). Glycogen was measured from ∼20 mg of liver using EnzyChrom Glycogen Assay Kit (BioAssay Systems), according to the manufacturer’s instructions.

### Plasma analyses

Plasma TG and total plasma cholesterol were measured from 2 and 10 μl of plasma, respectively, using Infinity Triglyceride and Cholesterol Reagents (Thermo Scientific). For high-density lipoprotein (HDL) cholesterol measurements, non-HDL was precipitated by incubating 20 μl of plasma with precipitation buffer containing 0.44 mM phosphotungstic acid and 20 mM MgCl_2_ for 10 min at room temperature, followed by centrifugation. Cholesterol was measured from the resulting supernatant. Plasma ALT, AST, bilirubin, ALP, albumin, and total protein were measured with Piccolo Liver Panel Plus discs used with a Piccolo Xpress chemistry analyzer (Abaxis).

### Statistical analyses

Results are expressed as mean ± standard deviation. Statistical significance was evaluated with unpaired Student two-tailed *t* test (if data passed a Shapiro-Wilk test for normality) or a Mann–Whitney *U* test (for nonparametric data, which did not pass test for normality). For experiments with multiple readouts, statistical significance was evaluated with two-way analysis of variance (ANOVA) with post hoc Šidák test, or repeated-measures ANOVA for time course experiments. Analyses were performed using GraphPad Prism 7. ∗*p* < 0.05, ∗∗*p* < 0.01, ∗∗∗*p* < 0.001, ∗∗∗∗*p* < 0.0001.

## Data availability

The mass spectrometry proteomics data have been deposited to the ProteomeXchange Consortium *via* the PRIDE partner repository (37) with the dataset identifier PXD033884. The mass spectrometry lipidomics data are available at the NIH Common Fund's National Metabolomics Data Repository (NMDR) website, the Metabolomics Workbench, https://www.metabolomicsworkbench.org, where it has been assigned Project ID PR001476.

## Supporting information

This article contains supporting information.

## Conflict of interest

T. C. W. is an investigator of the Howard Hughes Medical Institute.
