# Supplementary material for: Fitm2 is required for ER homeostasis and normal function of murine liver
Source: J Biol Chem. 2023 Feb 15;299(3):103022. doi: 10.1016/j.jbc.2023.103022 (PMC10027564; doi:10.1016/j.jbc.2023.103022)
Supplement: Supporting Tables S1–S4 and Figures S1–S4 [file mmc1.pdf]

Table S1. Mass spectrometry measurement of FIT1 and FIT2 in liver and skeletal muscle.

ID	Flox 1	Flox 2	Flox 3	Flox 4	FIT2-LKO 1	FIT2-LKO 2	FIT2-LKO 3	FIT2-LKO 4	FIT2-LKO 5	Flox 1
Tissue	Liver	Liver	Liver	Liver	Liver	Liver	Liver	Liver	Liver	Skeletal Muscle
FITM1	n.d.	n.d.	n.d.	n.d.	n.d.	n.d.	n.d.	n.d.	n.d.	26.0766
FITM2	26.4376	23.3978	21.1139	21.6667	n.d.	n.d.	n.d.	n.d.	n.d.	n.d.
ACTB	33.1945	34.6911	33.1693	32.9812	33.3333	32.6851	32.7691	33.077	33.0558	29.4254
FASN	33.8659	34.3608	33.6764	33.347	34.1397	33.5568	33.5418	33.7423	34.2724	24.6743
MYH2	n.d.	n.d.	n.d.	n.d.	n.d.	n.d.	n.d.	n.d.	n.d.	33.2602
MYH3	n.d.	n.d.	n.d.	n.d.	n.d.	n.d.	n.d.	20.3023	n.d.	32.9784

Table S2. Hepatic acyl-CoA measurements. Absolute and relative amounts of CoA and acyl-CoA species in Flox and FIT2-LKO chow livers, as measured by mass spectrometry. n=6/genotype. *q<0.05.

	nmol/g liver				relative abundance				q-value
	AVE		SD		AVE		SD		
	Flox	LKO	Flox	LKO	Flox	LKO	Flox	LKO	
**free CoA**	65.3	57.8	6.67	6.23	1.00	0.89	0.10	0.10	0.123
**acetyl**	79.5	71.2	10.7	6.21	1.00	0.89	0.14	0.08	0.191
**propionyl**	3.42	2.75	0.95	0.64	1.00	0.80	0.28	0.19	0.211
**crotonyl**	1.00	1.01	0.15	0.08	1.00	1.01	0.15	0.08	0.788
**butyryl**	5.64	6.70	1.01	1.12	1.00	1.19	0.18	0.20	0.174
**b-hydroxy propionyl**	0.47	0.51	0.06	0.08	1.00	1.09	0.13	0.17	0.788
**3-me-crotonyl**	0.53	0.52	0.17	0.14	1.00	0.98	0.31	0.27	0.788
**isovaleryl**	1.44	1.45	0.50	0.41	1.00	1.01	0.35	0.29	0.783
**malonyl/hydroxybutyryl**	7.62	9.01	1.92	1.23	1.00	1.18	0.25	0.16	0.204
**succinyl**	0.41	0.47	0.08	0.11	1.00	1.16	0.20	0.26	0.428
**itaconyl**	0.31	0.46	0.14	0.16	1.00	1.48	0.45	0.53	0.196
**glutaryl**	0.66	0.97	0.16	0.13	1.00	1.46	0.25	0.19	0.015*
**hexenoyl**	0.48	0.60	0.10	0.08	1.00	1.25	0.21	0.17	0.259
**hexanoyl**	1.97	3.12	0.52	0.78	1.00	1.59	0.26	0.40	0.027*
**8:00**	0.84	1.44	0.21	0.48	1.00	1.71	0.25	0.57	0.034*
**10:0**	0.81	0.87	0.19	0.17	1.00	1.07	0.23	0.21	0.608
**12:1**	0.37	0.31	0.13	0.04	1.00	0.84	0.34	0.11	0.555
**12:0**	0.49	0.40	0.15	0.07	1.00	0.81	0.30	0.15	0.264
**14:2**	1.15	0.82	0.55	0.20	1.00	0.72	0.48	0.18	0.264
**14:0**	1.21	1.24	0.35	0.14	1.00	1.03	0.29	0.11	0.783
**14:1**	1.02	1.13	0.50	0.29	1.00	1.11	0.49	0.29	0.608
**16:0**	4.84	7.00	0.93	1.20	1.00	1.45	0.19	0.25	0.017*
**16:1**	4.33	6.08	0.73	1.81	1.00	1.41	0.17	0.42	0.098
**18:0**	2.99	5.01	0.60	0.49	1.00	1.68	0.20	0.17	0.001*
**18:1**	8.70	19.86	1.23	5.05	1.00	2.28	0.14	0.58	0.002*
**18:2**	8.92	9.94	1.05	1.24	1.00	1.12	0.12	0.14	0.204
**18:3**	1.52	1.25	0.14	0.25	1.00	0.82	0.09	0.17	0.098
**19:0**	0.95	1.60	0.26	0.25	1.00	1.68	0.28	0.26	0.005*
**19:1**	0.70	0.87	0.19	0.11	1.00	1.24	0.27	0.15	0.123
**20:0**	1.27	1.73	0.32	0.11	1.00	1.36	0.25	0.09	0.017*
**20:1**	1.84	5.85	0.47	1.48	1.00	3.18	0.26	0.80	0.001*
**20:2**	1.49	3.16	0.51	0.40	1.00	2.12	0.34	0.27	0.001*
**20:3**	0.95	1.50	0.14	0.15	1.00	1.57	0.15	0.16	0.001*
**20:4**	2.74	2.56	0.29	0.35	1.00	0.93	0.10	0.13	0.377
**20:5**	1.21	1.83	0.11	0.22	1.00	1.51	0.09	0.18	0.001*
**21:0**	0.36	0.66	0.08	0.10	1.00	1.82	0.23	0.26	0.001*
**21:1**	0.26	0.37	0.05	0.06	1.00	1.40	0.18	0.24	0.017*
**22:1**	3.21	3.45	0.91	0.80	1.00	1.07	0.28	0.25	0.608
**22:2**	0.80	1.21	0.16	0.24	1.00	1.51	0.20	0.30	0.017*
**22:5**	0.45	0.60	0.05	0.05	1.00	1.32	0.11	0.12	0.002*
**22:6**	1.61	1.56	0.17	0.13	1.00	0.97	0.11	0.08	0.759

Table S3. Hepatic HFD acyl-CoA measurements. Absolute and relative amounts of CoA and acyl-CoA species in Flox and FIT2-LKO livers from HFD cohort, as measured by mass spectrometry. n=9/genotype. *q<0.05.

	nmol/g liver				relative abundance				q-value
	AVE		SD		AVE		SD		
	Flox	LKO	Flox	LKO	Flox	LKO	Flox	LKO	
**Free CoA**	65.2	97.1	10.32	12.25	1.00	1.49	0.16	0.19	<0.001*
**Acetyl**	41.9	28.5	8.46	7.21	1.00	0.68	0.20	0.17	0.005*
**Propionyl**	1.52	3.65	0.56	0.64	1.00	2.40	0.37	0.42	<0.001*
**Crotonyl**	0.40	0.26	0.19	0.08	1.00	0.63	0.46	0.21	0.053
**Butyryl**	2.23	3.09	1.23	1.55	1.00	1.38	0.55	0.70	0.196
**b-hydroxy propionyl**	0.17	0.23	0.08	0.12	1.00	1.34	0.48	0.66	0.196
**3-Me-Crotonyl**	0.09	0.15	0.03	0.08	1.00	1.72	0.33	0.85	0.046*
**Isovaleryl**	0.66	1.25	0.25	0.34	1.00	1.89	0.38	0.51	0.002*
**Malonyl/Hydroxybutyrl**	2.47	3.37	1.23	1.59	1.00	1.37	0.50	0.64	0.183
**Succinyl**	0.19	0.26	0.06	0.08	1.00	1.34	0.31	0.41	0.079
**Itaconyl**	0.21	0.23	0.11	0.10	1.00	1.09	0.51	0.47	0.509
**Glutaryl**	0.25	0.24	0.14	0.05	1.00	0.98	0.58	0.22	0.614
**Hexenoyl**	0.16	0.13	0.07	0.04	1.00	0.80	0.43	0.26	0.261
**Hexanoyl**	1.09	0.99	0.59	0.57	1.00	0.91	0.54	0.53	0.509
**8:0**	0.76	0.60	0.39	0.25	1.00	0.79	0.51	0.33	0.253
**10:0**	0.95	0.83	0.24	0.15	1.00	0.88	0.26	0.16	0.201
**12:0**	0.56	0.45	0.16	0.12	1.00	0.80	0.29	0.21	0.098
**12:1**	1.30	0.88	0.22	0.15	1.00	0.68	0.17	0.12	<0.001*
**14:0**	1.61	1.33	0.46	0.25	1.00	0.83	0.29	0.15	0.134
**14:1**	2.32	1.42	0.70	0.21	1.00	0.61	0.30	0.09	0.009*
**14:2**	0.77	0.49	0.12	0.10	1.00	0.64	0.16	0.13	<0.001*
**16:0**	4.26	4.27	1.47	1.40	1.00	1.00	0.34	0.33	0.668
**16:1**	8.50	4.15	2.43	0.41	1.00	0.49	0.29	0.05	0.002*
**18:0**	1.64	2.55	0.35	0.73	1.00	1.56	0.21	0.45	0.013*
**18:1**	18.90	13.92	4.10	2.46	1.00	0.74	0.22	0.13	0.013*
**18:2**	3.43	2.51	0.71	0.19	1.00	0.73	0.21	0.06	0.009*
**18:3**	0.33	0.25	0.08	0.04	1.00	0.74	0.24	0.11	0.020*
**19:0**	1.46	0.90	0.23	0.25	1.00	0.61	0.15	0.17	<0.001*
**19:1**	0.47	0.50	0.08	0.08	1.00	1.06	0.18	0.18	0.406
**20:0**	0.74	0.52	0.16	0.38	1.00	0.71	0.21	0.52	0.137
**20:1**	2.90	2.83	0.74	0.73	1.00	0.98	0.26	0.25	0.584
**20:2**	0.88	0.70	0.15	0.16	1.00	0.80	0.17	0.18	0.032*
**20:3**	0.58	0.90	0.12	0.13	1.00	1.56	0.22	0.22	<0.001*
**20:4**	1.36	2.21	0.20	0.38	1.00	1.62	0.15	0.28	<0.001*
**20:5**	0.24	0.32	0.03	0.03	1.00	1.38	0.13	0.12	<0.001*
**21:0**	0.86	0.36	0.19	0.11	1.00	0.42	0.22	0.12	<0.001*
**21:1**	0.20	0.17	0.04	0.03	1.00	0.84	0.22	0.15	0.079
**22:1**	1.73	0.57	0.50	0.19	1.00	0.33	0.29	0.11	<0.001*
**22:2**	0.40	0.23	0.10	0.08	1.00	0.58	0.25	0.21	0.003*
**22:5**	0.13	0.11	0.04	0.03	1.00	0.81	0.31	0.26	0.132
**22:6**	0.63	0.68	0.07	0.09	1.00	1.09	0.11	0.15	0.201

Table S4. qPCR primer sequences.

Gene name	F/R	Sequence
18S	F	CGCTTCCTTACCTGGTTGAT
18S	R	GAGCGACCAAAGGAACCATA
Acly	F	AAAGCTTGGCCTCGTCGG
Acly	R	GGGACGAAGGGTTCAATGAGA
Aox	F	CTCATCTTCGAGGCTTGGAAACCAC
Aox	R	ATTTCACGGATAGGGACAAGAAAGGC
Arbp	F	TCACTGTGCCAGCTCAGAAC
Arbp	R	AATTTCAATGGTGCCTCTGG
Atf3	F	GAGGATTTTGCTAACCTGACACC
Atf3	R	TTGACGGTAACTGACTCCAGC
Atp5a1	F	TCCATGCCTCTAACACTCGAC
Atp5a1	R	GACGTGTCAGCTCCCAGAA
Bax	F	GGAGATGAACTGGACAGCAATA
Bax	R	GAAGTTGCCATCAGCAAACAT
Bcl2	F	GAGCAGGTGCCTACAAGAAA
Bcl2	R	CTTTGTCCTCTGACTGGGTATG
Bip	F	ACTTGGGGACCACCTATTCCT
Bip	R	ATCGCCAATCAGACGCTCC
Chop	F	CCACCACACCTGAAAGCAGAA
Chop	R	AGGTGAAAGGCAGGGACTCA
Chrebpa	F	CGACACTCACCCACCTCTTC
Chrebpa	R	TTGTTCAGCCGGATCTTGTC
Chrebpb	F	TCTGCAGATCGCGTGGAG
Chrebpb	R	CTTGTCCCGGCATAGCAAC
Co1	F	CCCAATCTCTACCAGCATC
Co1	R	GGCTCATAGTATAGCTGGAG
Cox4i1	F	TCACTGCGCTCGTTCTGAT
Cox4i1	R	CGATCGAAAGTATGAGGGATG
Cpt1a	F	GAA CCC CAA CAT CCC CAA AC
Cpt1a	R	TCC TGG CAT TGT CCT GGA AT
Cre	F	CGGTCTGGCAGTAAAAACTAT
Cre	R	CAGGGTGTTATAAGCAATCCC
Cre	F	CCCTGTTTCACTATCCAGGT

Cre	R	GGGTAACTAAACTGGTCGAG
Cyclo	F	TGGAGAGCACCAAGACAGACA
Cyclo	R	TGCCGGAGTCGACAATGAT
Dgat1	F	GGAATATCCCCGTGCACAA
Dgat1	R	CATTTGCTGCTGCCATGTC
Dgat2	F	CCGCAAAGGCTTTGTGAA
Dgat2	R	GGAATAAGTGGGAACCAGATCAG
Edem	F	AAGCCCTCTGGAACCTTGCG
Edem	R	AACCCAATGGCCTGTCTGGT
F4/80	F	TGACTCACCTTGTGGTCCTAA
F4/80	R	CTTCCCAGAATCCAGTCTTTCC
Fitm2	F	CGAGAGCTACCTCAGCAACAAG
Fitm2	R	CAATGAAAGGCAGGAGGAGACA
H19	F	GTACCCACCTGTCGTCC
H19	R	GTCCACGAGACCAATGACTG
Hmgcr	F	CTTGTGGAATGCCTTGTGATTG
Hmgcr	R	AGCCGAAGCAGCACATGAT
Hmgcs	F	GCCGTGAACTGGGTCGAA
Hmgcs	R	GCATATATAGCAATGTCTCCTGCAA

Figure S1

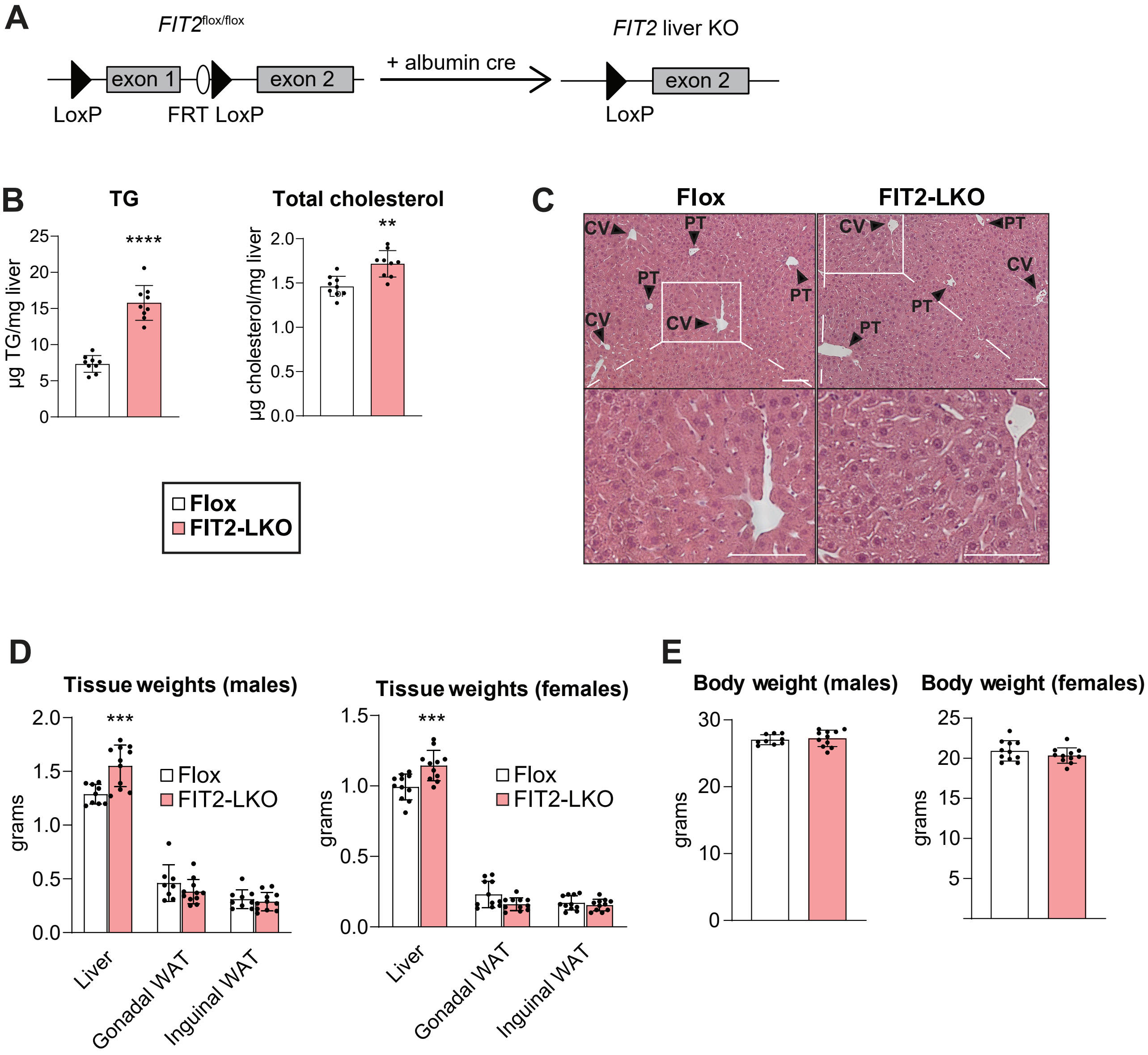

Figure S1. Corresponding to Figure 1. (A) Genetic construct used for generation of murine model of FIT2 deficiency in hepatocytes. (B) Liver-specific FIT2 deficiency elevates liver TG and total cholesterol in female mice (n=9/genotype). (C) Representative images of H&E staining of livers from male Flox and FIT2-LKO mice. Scale bar=50 μm. CV=central vein; PT=portal triad. (D) FIT2-LKO mice have heavier livers than flox littermates. (E) FIT2-LKO mice do not exhibit altered body weight, compared to flox control littermates with chow feeding (n=9-11/genotype). Data represents mean ± SD. Statistical significance was evaluated with unpaired Student's 2-tailed t-test (B, E) and 2-way ANOVA with Šidák correction in (D). *p<0.05, **p<0.01, ***p<0.001, ****p<0.0001.

Figure S2

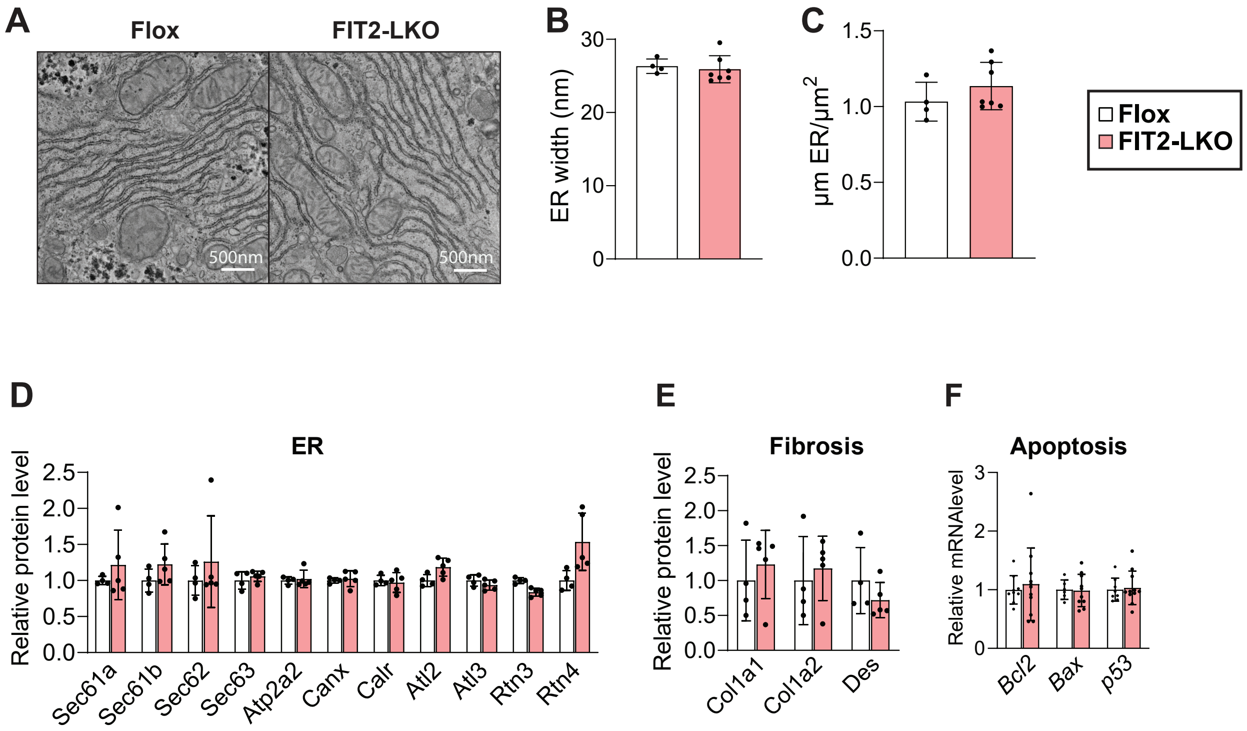

Figure S2. Corresponding to Figure 2. Representative electron microscopy images (A) and quantification of ER dilation (B) indicate that loss of FIT2 does not alter ER ultrastructure in hepatocytes (n=5–8). Flox and FIT2-LKO hepatocytes contain equal amounts of ER, as quantified from electron microscopy images (n=5–8/genotype) (C). Protein abundance of ER proteins (D) and fibrosis markers (E), as measured by mass spectrometry (n=4–5/genotype). (F) FIT2-LKO mice do not exhibit evidence for apoptosis; transcript levels of anti-apoptotic (Bcl2) and pro-apoptotic (Bax, p53) gene markers are unaltered (n=9–11/genotype). Data represents mean ± SD. Statistical significance for (B, C, F) was evaluated with unpaired Student's 2-tailed t-test. Statistical significance for the proteomics dataset (D, E) was calculated using a Student's t-test followed by Benjamini-Hochberg FDR correction of 5%. $^{*}p<0.05$, $^{**}p<0.01$, $^{***}p<0.001$.

Figure S3

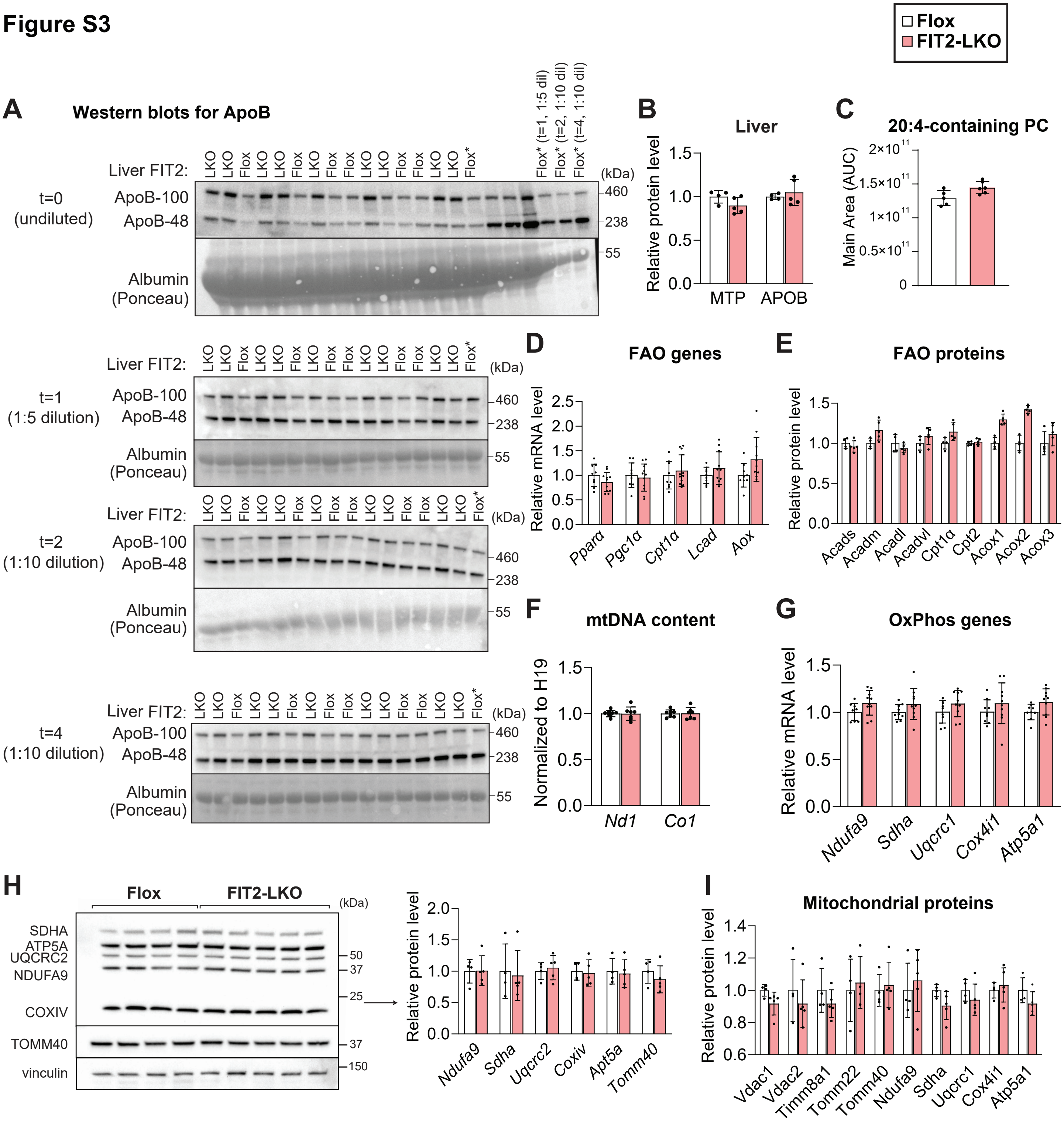

Figure S3. Corresponding to Figure 3. (A) Immunoblotting of plasma apoB-100 and apoB-48 used for quantification of ApoB secretion in Figure 5A. (B) Proteomic measurements indicate that amounts of APOB and MTTP protein levels are not altered in the liver between Flox and FIT2-LKO mice (n=4–5/genotype). (C) Levels of 20:4-containing phosphatidylcholine are not significantly altered between genotypes, as assessed by mass spectrometry. Genes (D) and protein (E) involved in fatty acid oxidation (FAO) are not reduced in FIT2-LKO livers (n=9–11/genotype). (F) Levels of hepatic mitochondrial (mt) DNA do not differ between genotypes. OxPhos genes (G) and proteins (H, I) are not altered in FIT2-LKO livers. Data represents mean ± SD. Statistical significance for (C, D, F, G, H) was evaluated with unpaired Student's 2-tailed t-test. Statistical significance for the proteomics dataset (E, I) was calculated using a Student's t-test followed by Benjamini-Hochberg FDR correction of 5%.

Figure S4.

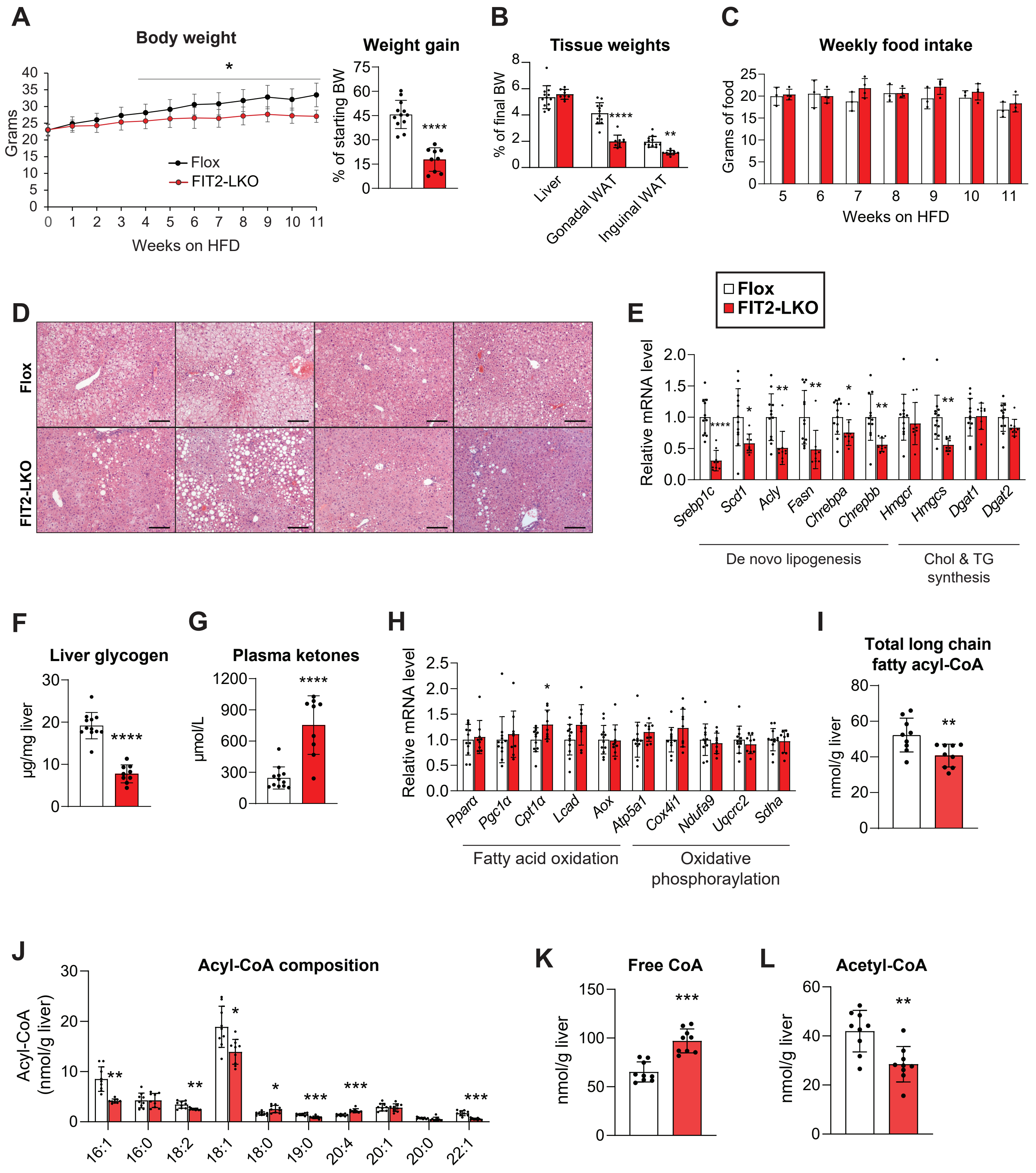
A
Body weight
Grams
Weeks on HFD
Flox
FIT2-LKO
Weight gain
% of starting BW
B
Tissue weights
% of final BW
Liver
Gonadal WAT
Inguinal WAT
C
Weekly food intake
Grams of food
Weeks on HFD
D
Flox
FIT2-LKO
E
Flox
FIT2-LKO
Relative mRNA level
Srebp1c
Scd1
Acly
Fasn
Chrebpa
Chrepbb
Hmgcr
Hmgcs
Dgat1
Dgat2
De novo lipogenesis
Chol & TG synthesis
F
Liver glycogen
μg/mg liver
G
Plasma ketones
μmol/L
H
Relative mRNA level
Pparα
Pgc1α
Cpt1α
Lcad
Aox
Atp5a1
Cox4i1
Ndufa9
Uqcrc2
Sdha
Fatty acid oxidation
Oxidative phosphoraylation
I
Total long chain fatty acyl-CoA
nmol/g liver
J
Acyl-CoA composition
Acyl-CoA (nmol/g liver)
16:1
16:0
18:2
18:1
18:0
19:0
20:4
20:1
20:0
22:1
K
Free CoA
nmol/g liver
L
Acetyl-CoA
nmol/g liver

Figure S4. **Corresponding to Figure 4.** FIT2-LKO animals weigh significantly less (A) and have reduced adiposity (B) in both gonadal (gWAT) and inguinal (iWAT) white adipose depots compared to Flox mice after an 11-week high-fat diet (HFD) feeding study. (C) Food intake is not different between genotypes. Animals were individually caged and food consumption was measured weekly for 7 weeks (n=3-4/genotype). (D) Additional images of H&E staining of livers from Flox and FIT2-LKO mice after HFD challenge (n=5-9/genotype). Scale bar=50 μm. (E) Under HFD feeding, FIT2-LKO mice exhibit decreased expression of genes related to de novo lipogenesis and cholesterol synthesis. They also show a decrease in liver glycogen content (F) and an increase in plasma ketone bodies (KB) (G). The FIT2-LKO mice exhibit little to no change in gene markers for fatty acid oxidation or oxidative phosphorylation (H). (I) FIT2-LKO livers contain elevated levels of total long-chain fatty acids (≥16 carbon length). (J) Amounts of individual long-chain acyl-CoA species in livers of Flox and FIT2-LKO mice. Levels of hepatic free CoA (K) were elevated in the FIT2-LKO mice, while acetyl-CoA (L) levels were decreased. Data represents mean ± SD. N=9–12/genotype, unless otherwise noted above for specific experiments. Statistical significance for body weight over time (A, left) was evaluated with repeated-measures ANOVA and the weight gain (A, right) was evaluated using an unpaired Student’s 2-tailed test. (B) and (C) were evaluated with 2-way ANOVA with Šidák correction. For (E), statistical significance was evaluated with unpaired Student’s 2-tailed t-test for parametric data and a Mann-Whitney U test for nonparametric data. Statistical significance for (F-I) were evaluated with Student's 2-tailed t-test, and (J-L) were evaluated with multiple unpaired t-tests with a False Discovery Rate (FDR) of 1%. *$p<0.05$, **$p<0.01$, ***$p<0.001$, ****$p<0.0001$ (for J-L, the q-value is reported).
